# The Associations Between Physical Activity and Gut Microbiota Among Older Community-Dwelling Men

**DOI:** 10.1093/geroni/igaa057.3075

**Published:** 2020-12-16

**Authors:** Lisa Langsetmo, Abigail Johnson, Ryan Demmer, Kristine Ensrud, Eric Orwoll, James Shikany

**Affiliations:** 1 University of Minnesota, Minneapolis, Minnesota, United States; 2 Oregon Health & Science University, Portland, Oregon, United States; 3 University of Alabama at Birmingham, Birmingham, Alabama, United States

## Abstract

We determined the relationship between objectively measured physical activity (PA) and the gut microbiome among community-dwelling older men from the Osteoporotic Fractures in Men (MrOS) cohort participants at Visit 4 (2014-16). Eligible men (n=373, mean age 84 y) included participants with 5-day activity assessment and stool samples analyzed for 16S marker genes. Armband data together with sex, height, and weight were used to estimate total steps and energy expenditure. We used linear regression analysis, principal coordinate analysis, zero-inflated Gaussian models to assess association between PA and α-diversity, β-diversity, and specific taxa, respectively, with adjustments for age, race, BMI, clinical center, library size, and number of chronic conditions. There was a slight association between PA and β-diversity but no association with α-diversity. After multivariate adjustment, those who had higher step counts vs lower step counts had higher relative abundance of Cetobacterium and lower relative abundance of Coprobacillus, Adlercreutzia, Erysipelotrichaceae CC-115.

